# Rheumatoid arthritis prevention: any takers?

**DOI:** 10.1136/rmdopen-2021-001633

**Published:** 2021-04-08

**Authors:** Marie Falahee, Karim Raza

**Affiliations:** 1 Rheumatology Research Group, Institute of Inflammation and Ageing, University of Birmingham College of Medical and Dental Sciences, Birmingham, UK; 2 Rheumatology Department, Sandwell and West Birmingham Hospitals NHS Trust, Birmingham, UK; 3 NIHR Birmingham Biomedical Research Centre, University Hospitals Birmingham NHS Foundation Trust and University of Birmingham, Birmingham, UK; 4 MRC Versus Arthritis Centre for Musculoskeletal Ageing Research and the Research into Inflammatory Arthritis Centre Versus ArthritisBirmingham, University of Birmingham, Birmingham, UK

**Keywords:** arthritis, arthritis, rheumatoid, psychology

Our understanding of biological mechanisms operating at articular and extra-articular sites in individuals ‘at risk’ of rheumatoid arthritis (RA) has increased significantly over recent years.^1^ In parallel, there has been significant progress in the prediction of RA development in those at risk.^2^ This has opened up an agenda for research on possibilities for intervention in pre-RA phases, and opportunities for both primary and secondary prevention have been identified.^3–5^

Intervention at the very earliest stages of disease development could, in theory, control symptoms such as arthralgia and fatigue that often precede the development of clinical arthritis,^6^ delay the onset of RA, reduce the likelihood of RA developing and/or reduce the severity of RA if it were to develop. While the evidence base to support such strategies is in its infancy, B-cell depletion, with a single infusion of 1000 mg of rituximab, has been shown to significantly delay the onset of RA in individuals with autoantibody-positive arthralgia and either an inflammatory response as measured by C-reactive protein or subclinical synovitis on imaging.^7^ Similarly, the impact of time-limited courses of other immunomodulatory therapies, including abatacept^8^ and hydroxychloroquine,^9^ on arthritis and RA development is being assessed in other at-risk groups. Results of these studies,^8 9^ where the intervention is given for 12 months, and other studies, where interventions are given for different but nevertheless time-limited durations, are awaited with interest. Preventive strategies are also under investigation in other chronic autoimmune conditions. For example, an anti-CD3 antibody has recently been shown to significantly delay progression to type 1 diabetes in non-diabetic relatives of patients identified as being at high risk on the basis of the presence of diabetes-related autoantibodies and other risk factors.^10^

To have clinical impact, therapeutic approaches identified as being effective in reducing the risk of RA need to be acceptable to those at risk. Previous qualitative research has identified that individuals at risk of RA have concerns about taking ‘preventive’ medicines.^11^ In this issue of *RMD Open*, van Boheemen *et al*^2^ report on the perspectives of individuals who had declined participation in, and also who had participated in, one of the two RA prevention trials: the STAtins to Prevent Rheumatoid Arthritis trial (RMD open to insert Ref) and the Arthritis Prevention In the Pre-clinical Phase of RA with Abatacept trial.^8^ Challenges to participant recruitment in trials of patients with seropositive arthralgia raise some important issues for the rheumatology research community to consider and reflect the importance of understanding the preferences of at-risk individuals about benefits and risks of interventions in relation to the disease they are at risk of.^12^

Public perceptions about RA are often inaccurate: many do not perceive RA to be a serious disease, and some view it as an inevitable consequence of ageing.^13 14^ Individuals with these views may be less likely to accept preventive therapeutic interventions—this was certainly a theme identified by van Boheemen *et al*^2^. Positive views about RA prediction and prevention are often associated with misperceptions around what being at ‘high risk’ means (eg, some interpret this to mean that they will definitely develop RA) and the likely benefit of ‘preventive’ therapy (eg, for some this means the therapy will definitely prevent them from developing RA).^15–17^ The development of effective communication tools around predictive testing, preventive interventions and RA itself is therefore essential to facilitate informed decision-making. Although predictive algorithms exist, they do not fully address issues around the time course of RA development or the likely severity of RA after it has developed, which are key considerations for decision-making about preventive therapy. Further research to facilitate comprehensive risk assessment for RA is an essential precursor to the development of effective informational resources for individuals for whom preventive treatment may be appropriate.

Non-pharmacological interventions are preferred by some at-risk individuals, especially for those without symptoms (eg, autoantibody-positive individuals and those with genetic risk factors)^11 15–19^ van Boheemen *et al*^2^. Initial evidence suggests that personalised risk information about RA has a positive impact on behavioural intentions and risk-reducing behaviour^20^ while providing reassurance to recipients.^21^ Although several ongoing studies are investigating pharmacological interventions to prevent arthritis development, no interventional trials of promising behavioural interventions, particularly smoking cessation,^22 23^ to reduce risk of RA development and progression have been published. Investigation in this area is needed.

Preventive therapies for other conditions are well established in routine clinical practice; statins and antihypertensive medications are widely prescribed to reduce the risk of ischaemic heart disease and bisphosphonates to reduce the risk of fracture. Many entirely asymptomatic individuals with conditions such as hypercholesterolaemia, hypertension and osteoporosis accept such pharmacological therapies. Indeed, for these conditions, long-term, often lifelong, preventive treatment is both required and often accepted. As yet, no pharmacological treatments have been shown to reduce the risk of RA development in the medium to long term. However, this remains an active and important area of research. We need to learn from experiences in other chronic diseases and address the barriers identified by van Boheemen *et al*^2^ that hinder the efficient development of preventive strategies for the management of RA. Endeavouring to overcome such barriers is likely to be worthwhile, given that a ‘preventive’ approach in RA has clear potential to reduce pain and disability as well as societal costs resulting from lost productivity and healthcare usage at huge scale.

## Data Availability

No additional data are available.
